# Maximizing Oviposition Efficiency when Mass Rearing the Coccinellid, *Sasajiscymnus tsugae,* a Predator of the Hemlock Woolly Adelgid, *Adelges tsugae*

**DOI:** 10.1673/031.010.14112

**Published:** 2010-09-14

**Authors:** Hugh E. Conway, Joseph D. Culin, LayLa W. Burgess, Cora Allard

**Affiliations:** ^1^ Pest Detection, Diagnostic & Management Laboratory, USDA-APHIS-PPQ, Edinburg, TX 78541; ^2^ Department of Entomology, Soils, and Plant Sciences, Clemson University, Clemson, SC 29634; ^3^ Department of Biological Sciences, Clemson University, Clemson, SC 29634

**Keywords:** biological control, invasive species

## Abstract

*Sasajiscymnus tsugae* Sasaji and McClure (Coleeptera: Coccinellidae), is a biological control agent imported for management of hemlock woolly adelgid, *Adelges tsugae* Annand. In mass rearing *S. tsugae,* accurate estimation of egg numbers is important because larvae are cannibalistic, especially at higher densities. To determine the most accurate means of estimating egg production, three brands of gauze were compared as oviposition substrates. Curad® gauze provided the most accurate estimate of egg production, and was the most cost effective brand. When eggs were collected from oviposition jars, similar adult yields of *S. tsugae* occurred between rearing cages infested with 1,650 eggs from gauze compared to eggs on the twigs from within these jars. Additionally, orientation of oviposition jars impacted *S. tsugae* egg production as significantly more eggs were produced in horizontally oriented oviposition jars.

## Introduction

The hemlock woolly adelgid, *Adelges tsugae* Annand, (Hemiptera: Adelgidae) was accidentally introduced to the eastern US from Japan in the early 1950s (Havill et al. 2006). *A. tsugae* is now considered a significant threat to both eastern hemlock, *Tsuga canadensis* L. (Pinales: Pinaceae) and Carolina hemlock (*T. caroliniana*) in the eastern United States (Knauer et al.2002). *A. tsugae* feeding causes both needle loss and bud death which can result in tree mortality in as little as four years (Cheah and McClure 1998; McClure et al. 2001).

In a forest environment, biological control can be an environmentally and economically effective method of managing *A. tsugae.* In the United States, many native predators occasionally feed on *A. tsugae,* but none significantly impact this pest which multiplies rapidly via parthenogenesis (Montgomery and Lyon 1996; Wallace and Hain 2000). The first non-native biological control agent released for *A. tsugae* management was *Sasajiscymnus tsugae* Sasaji and McClure (Coleoptera: Coccinellidae), which was introduced from Japan (Cheah and McClure 1998). Since 1997, *S. tsugae* has been mass reared and released as an important component of *A. tsugae* management programs (Cheah et al. 2005). When mass rearing *S. tsugae,* Palmer and Sheppard (2002) found that within oviposition containers, females would oviposit on both hemlock twigs and squares of gauze placed within *A. tsugae* infested hemlock bouquets. *Sasajiscymnus tsugae* eggs oviposited on gauze are easier to locate under microscopy than those on hemlock twigs, and provide a good estimate of total egg production. Accurate estimates of egg production are important because *S.* *tsugae* larvae cannibalize (Blumenthal 2002), and adult production decreases when >1,650 eggs are placed in a 61cm × 61cm × 49cm larval rearing chamber (Conway et al. 2005).

The objectives of this study were to determine the type of gauze that provided the most accurate and cost effective estimation of oviposition when mass rearing *S*. *tsugae,* and to examine whether oviposition jar orientation had an impact on the number of eggs produced by *S.* *tsugae.*

## Methods & materials

### Oviposition jar protocol

**Figure 1.**
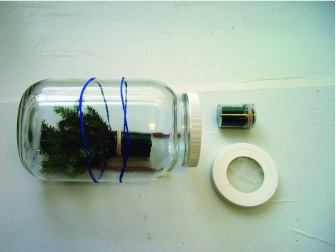
Glass (3.8 L) oviposition jar used in mass rearing *Sasajiscymnus tsugae* at Clemson University's insectary (Clemson, SC). Assembled jar shown at left, with hemlock bouquet inside, rubber bands holding two pencils underneath to prevent rolling, and lid in place. Plastic vial (6 cm tall × 4 cm diam) to hold the bouquet is shown at upper right, ventilation detail in lid shown at lower right. High quality figures are available online.

**Figure 2.**
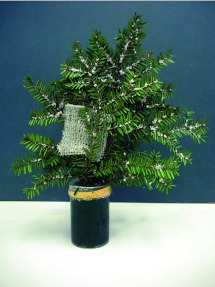
Hemlock bouquet. Only a single 5 cm × 5 cm gauze pad is shown for clarity. High quality figures are available online.

In these studies, oviposition jars (Figure 1) consisted of a 3.8 L glass jar with an 8 cm diameter hole cut in the plastic lid and covered with Noseeum netting (97 holes/cm^2^) (Equinox, www.equinoxltd.com). Each jar contained a bouquet of *A. tsugae* infested hemlock twigs (Figure 2). Bouquets were prepared by placing a water-soaked Wet Foam (FloraCraft www.floracraft.com) cylinder into a 6 cm tall by 4 cm diameter plastic vial. The open end of the vial was then covered by Cling Wrap (www.glad.com) which was secured with a rubber band. Six 20–25 cm long *A. tsugae* infested hemlock twigs were inserted through the Cling Wrap into the Wet Foam. Three 5 × 5 cm pieces of gauze were placed within each bouquet.

After bouquets were placed in jars, 10 female and 5 male sexually mature *S. tsugae* were added to each jar. In the rearing program bouquets were removed from oviposition jars weekly. Eggs on both gauze and twigs were transferred to larval rearing boxes, and all adults were returned to a jar containing a new bouquet. Missing or dead adults were replaced with beetles of the appropriate gender that were at least 30 days old. Cheah and McClure (1998) have shown that at 25° C, *S. tsugae* males and females reached maturity at approximately 19 and 22 days, respectively. All oviposition jars were maintained in a controlled-environment (25 ± 1°C, 60 ± 5% humidity, and 16:8 L:D) room.

### Effect of gauze type

Studies to determine whether *S*. *tsugae* exhibited a preference for oviposition on different brands of gauze under insectary conditions were conducted from 19–30 January (41 oviposition jars), 9–13 February (45 jars), and 16–20 February (58 jars) 2004. Three commonly available brands of gauze were examined. Both Curad® Basic Care (www.curadusa.com) and Kling® (Johnson & Johnson Consumer Companies, Inc., www.jnj.com) were cotton, while First Aid Gauze Pads (Johnson & Johnson) were rayon/cellulose. The three pieces of gauze placed within each hemlock bouquet consisted of one piece of each brand. To control for position effect on oviposition the location of each brand was randomly assigned to the base, middle, or tip of each bouquet. After 1 week hemlock bouquets were removed from oviposition jars and the numbers of viable *S. tsugae* eggs on each gauze brand were recorded for each container. Standard ANOVA procedures with Tukey-Kramer means comparisons for all pairs were used to determine oviposition differences among gauze brands (SAS 2006).

### Gauze cost analysis

Because cost of gauze varied among the three brands listed above, we examined the average cost of a 5 × 5 cm piece of gauze for each of these brands. Prices were obtained on 10 August 2004 at five local merchants (Wal-Mart, Kmart, Target, CVS Pharmacy, Eckerd Pharmacy) and three online retail sources (Amazon.com, Medico-school.com, and Westburypharmacy.com). Standard ANOVA procedures with Tukey-Kramer means comparisons were used to determine cost differences among gauze brands (SAS 2006).

### Effect of eggs on gauze vs eggs on twigs

**Figure 3.**
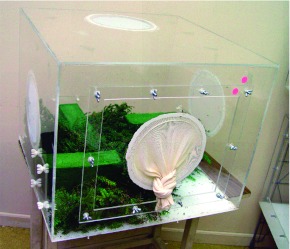
Plexiglass rearing chamber. Wet foam blocks hold hemlock twigs infested with *Adelges tsugae* that serve as food for larval *Sasajiscymnus tsugae.* High quality figures are available online.

Additional studies were conducted from 30 January to 23 June 2004 to determine the relationship between the number of *S. tsugae* eggs deposited on Curad® Basic Care gauze and the number of eggs deposited on hemlock twigs within individual oviposition jars. To do this 100 pairs of larval rearing chambers were established as in Conway et al. (2005). *S. tsugae* eggs were counted on each gauze pad from a group of oviposition jars until a total of 1,650 eggs was reached. These gauze pads were then placed into one larval rearing chamber (Figure 3) of a pair creating a known-number egg cohort. All hemlock twigs from those same oviposition jars were then placed into the other larval rearing chamber of the pair creating an unknown-number egg cohort. Larval rearing chambers were then maintained in a controlled-environment (25 ± 1° C; 60 ± 5% humidity; 16:8 L:D) room. After a 35 ± 3 day developmental period, the number of adult *S.* *tsugae* emerging within each chamber was recorded. The numbers of adults emerging from known-number (gauze) egg cohorts were compared to the numbers emerging from unknown-number (twig) egg cohorts using Matched Pairs with Wilcoxon Sign-Rank (SAS 2006).

### Effect of jar orientation

**Figure 4.**
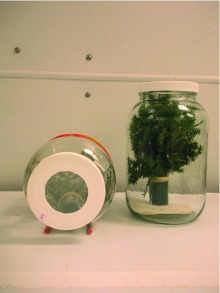
Oviposition jars shown in horizontal (left) and vertical (right) positions. High quality figures are available online.

Because oviposition jars require less shelf space if standing vertically (0.027 m^2^ / jar) than lying horizontally (0.093 m^2^ / jar), a study was conducted from 6 December 2004 to 14 January 2005 to determine if jar orientation had an impact on egg production. On each day of this study equal numbers of oviposition jars were prepared as described above, then randomly assigned to either the standard horizontal position or a vertical position (Figure 4). A total of 60 jars were assigned to each group per replication, three replications were conducted, and only Curad® gauze pads were used in this study. Hemlock bouquets were removed weekly from oviposition jars, and the numbers of viable *S. tsugae* eggs deposited on the Curad® gauze pads were recorded for each of the two orientations. Standard ANOVA procedures with Tukey-Kramer means comparisons for all pairs were used to determine oviposition differences between jar orientations (SAS 2006).

## Results and Discussion

### Effect of gauze type

*S.* *tsugae* females oviposited significantly more eggs on Curad® than First Aid Gauze* Pads (Table 1). Although not significant, egg numbers were higher on Curad® than Kling® in all replicates (Table 1). Egg numbers did not differ significantly between Kling® and First Aid Gauze* Pads (Table 1). The mean number of eggs laid per individual gauze pad increased as the experiment progressed through time with highest numbers occurring in the last trial. Although it was not quantified, both Curad® (cotton) and Kling® (cotton) were lighter in texture and had thinner thread diameter than the First Aid Gauze* Pads (rayon/cellulose). Seagraves (2009) found that coccinellids prefer to lay their eggs close to a food source, and in this study the white cotton gauze closely mimics the color of adelgid ovisacs. Additionally, it was observed that the loose weave on the cotton gauze allowed females to attach eggs in and among the cotton treads. On the more tightly woven rayon/cellulose pads eggs were only found to be attached to the edges of the pads. In most cases, eggs were attached primarily on the top and bottom edges of these pads.

### Gauze cost analysis

A single 5 × 5 cm piece of First Aid Gauze* Pad cost significantly more than both Kling® and Curad®, while Kling® cost significantly more than Curad® (Table 1). The higher number of eggs laid on Curad® gauze reduces handling time when counting eggs on gauze. Additionally, Curad® gauze cost $5.10 per day less than Kling® gauze when 100 rearing jars are in production providing a substantial cost savings of over $700.00 per year compared to other readily available gauze materials

### Effect of eggs on gauze vs eggs on twigs

The mean number of adults emerging per larval rearing box varied throughout the rearing season with highest adult production (> 825 adults per box) occurring in larval rearing boxes established between 2 February and 25 March (adults harvested between 12 March and 29 April). Palmer and Sheppard (2000) have shown that *S. tsugae* development is maximized when feeding on host eggs, and McClure (1989) reported that this is when *A. tsugae* systens egg production is greatest.

The largest number of *S. tsugae* adults produced from eggs on twigs in a single rearing box was 1207 adults on 2 February and from eggs on Curad® gauze was 1308 adults on 24 Feb. There was no significant difference between the mean numbers of *S. tsugae* adults emerging per rearing chamber across the season when starting with eggs on Curad® gauze (0 = 399.7 ± 14.7 (SEM)) compared to eggs on hemlock twigs (0 = 395.2 ± 14.7 (SEM)) (t_1,99_ = - 0.30, [t] = 0.76, Correlation 0.77). Estimating that there is a 1:1 ratio of eggs on gauze to eggs on twigs allows rearing facility technicians to easily and accurately approximate the total number of eggs placed in larval rearing chambers. Palmer and Sheppard (2002) reported an approximate 1:1 ratio of *S.* *tsugae* eggs deposited on gauze and twigs in 3.8L glass jars, but did not report on the type of gauze used. The data from this study proved that use of Curad® gauze as an oviposition substrate is an effective means to efficiently gather approximately 1650 eggs to place in a larval rearing chamber.

**Table 1.**
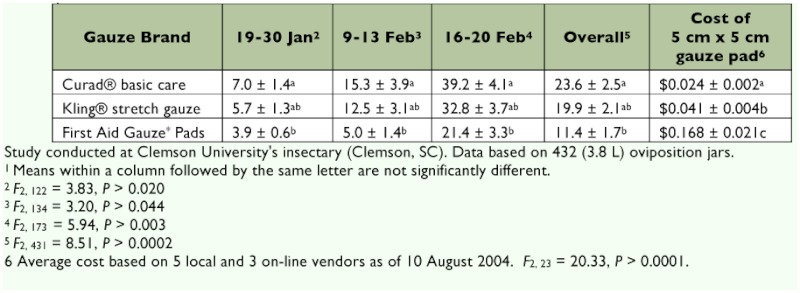
Mean^1^ ± SEM number of *Sasajiscymnus tsugae* eggs deposited on 5 cm × 5 cm gauze pads, from 19 January to 20 February 2004.

Adult adelgids aestivate from August to December in forest situations, and are poor quality food for *S. tsugae* (Cheah and McClure 2000). To ensure use of the highest quality host material possible we began collecting at relatively low elevations in South Carolina and Georgia in December as *A. tsugae* broke aestivation, and progressed to higher elevations in North Carolina by the end of the rearing cycle in June as they entered aestivation. Sightly higher numbers of *S. tsugae* emerged from larval rearing containers containing *A. tsugae* eggs on twigs when host quality was poorest and slightly more *S. tsugae* emerged from containers with *A. tsugae* eggs on gauze when host quality was highest.

### Effect of jar orientation

The number of *S. tsugae* eggs on gauze in oviposition jars maintained in a horizontal position was found to be significantly greater than in jars maintained in a vertical position in the second and third replications as well as in the overall data (Table 2). The orientation of hemlock twigs in horizontal oviposition jars was similar to the twig arrangement found naturally on hemlock trees. In all replications more eggs were laid in jars held in the horizontal position than the vertical position. When *S. tsugae* adults were first placed into rearing jars they tended to move around the sides and lid of the container. However, no eggs were found on the noseeum netting on the jar lids. Once adults had settled onto the twigs with *A. tsugae,* the predators did little wandering.

In the laboratory, 100 horizontally positioned oviposition jars were typically maintained each week. Based on oviposition data, it would require an average of 117 vertical jars to equal the egg production of 100 horizontal jars. Although vertical jar placement would require approximately 1/3 the total shelf space (9.3 m^2^ for horizontal versus 3.16 m^2^ for vertical), vertical placement would also require 17% more staff time to recover eggs from the additional jars.

## Conclusions

Successful establishment of *S. tsugae* as a biological control agent for HWA relies on development of economically efficient rearing techniques that maximize production. When using quality *A. tsugae* as the food source, slight modifications to the initial & *tsugae* rearing techniques presented by Palmer and Sheppard (2002) can increase the number of adult *S. tsugae* produced while reducing production costs. Techniques developed for mass rearing *S. tsugae* should provide a solid base for rearing similar coleopteran biological control agents for use in HWA management.

**Table 2.**
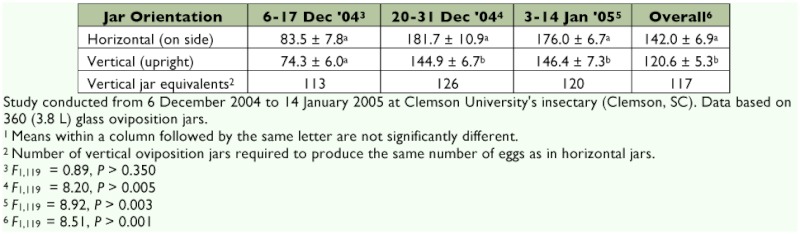
*Sasajiscymnus tsugae* mean^1^ ± SEM egg production based on oviposition jar orientation.
